# An Investigation on Internal Material Loads and Modifications in Precision Turning of Steel 42CrMo4

**DOI:** 10.3390/mi12050526

**Published:** 2021-05-06

**Authors:** Tjarden Zielinski, Andrey Vovk, Oltmann Riemer, Bernhard Karpuschewski

**Affiliations:** 1 Leibniz-Institut für Werkstofforientierte Technologien—IWT, 28359 Bremen, Germany; vovk@iwt.uni-bremen.de (A.V.); riemer@iwt.uni-bremen.de (O.R.); karpuschewski@iwt-bremen.de (B.K.); 2 MAPEX Center for Materials and Processes, University of Bremen, 28359 Bremen, Germany

**Keywords:** process signatures, material loads, material modifications, precision machining, steel

## Abstract

The functional properties of a workpiece are determined by a modification of the surface and subsurface materials. In this work, the correlation between thermo-mechanical material loads and the modification of the residual stresses is presented. While the resulting residual stresses were measured by X-ray diffraction after machining experiments, the material loads were determined using a process simulation. The experimental data (measured process forces and results from previous experiments) are used to validate the simulation, which is then applied to calculate the internal thermo-mechanical loads of the maximal temperature and the equivalent von-Mises-stresses per volume element during the machining experiments. In conclusion, a higher depth impact of mechanical loads compared to a lower depth impact of thermal loads in precision machining is observed. For the sake of novelty, the thermo-mechanical loads were plotted and interpreted in a three-dimensional fashion. Finally, cross sections of this mutual representation at certain constant material loads—thermal and mechanical—result in a process signature, which can prospectively improve the prediction of functional workpiece properties.

## 1. Introduction

High-precision milling and cutting are well-established machining processes in industrial production, and much research has been conducted in this area. In the context of the quality of the achieved machining result, the state of the surface and subsurface layer after machining is referred to as surface integrity [1]. The surface integrity of machined parts has long been improved regarding dimensional accuracy or surface roughness. Even subsurface properties such as residual stresses or the hardness can be altered with machining processes. However, the exact mechanisms and the impact of local thermo-mechanical loads on the surface and subsurface material properties are often uncertain before machining. Therefore, desired properties can only be achieved with subsequent adjustment of the process parameters and analysis in between. In order to avoid these laborious and tedious methods, it is necessary to understand and describe mechanisms of material modification with respect to machining. The interdisciplinary Transregional Collaborative Research Center (SFB TRR) 136 “Process Signatures” strives to investigate the process loads and material modification mechanisms in various machining processes in order to predict surface integrity [2,3,4]. Prior to this investigation, the material model was validated regarding temperature development and process forces in fly-cutting experiments [5]. In the investigation presented here, the local thermo-mechanical material loads are determined in the process simulation of machining experiments with a coupled Eulerian–Lagrangian (CEL) simulation. This simulation method was used for modelling orthogonal cutting [6] and chip formation [7] in the literature and has also been proven to offer good results in the determination of residual stresses [8], temperature development and plastic strain [9]. For the sake of novelty in this investigation, the results are correlated with the material modification of the residual stresses to determine a process signature and to improve the prediction of the surface and subsurface properties of machined workpieces.

## 2. Materials and Methods

### 2.1. Workpiece Material

In this investigation, cylindrical workpiece specimens with a diameter (Ø) of 58 mm and a 1 mm wide ring on the face were used. The workpiece material is steel 42CrMo4 (AISI 4140) heat-treated and tempered to a hardness of 42 HRC. In order to achieve this hardness, an austenitization temperature of 850 °C and a tempering temperature of 475 °C with quenching in oil in between were applied after pre-machining the specimen. The chemical composition of the workpiece material is displayed in Table 1.

### 2.2. Cutting Tools

The cutting tools used in the experiments were insert tools RS274.0200.06.M0 TH35 (Hartmetall-Werkzeugfabrik Paul Horn GmbH, Tuebingen, Germany) made of cemented carbide with a TiAIN coating. The inserts possess a straight cutting edge with a width of 2 ± 0.02 mm, a corner edge radius of 0.2 mm, a rake angle of 0° and a clearance angle of 7°.

### 2.3. Experimental Procedure

**Figure 1:**
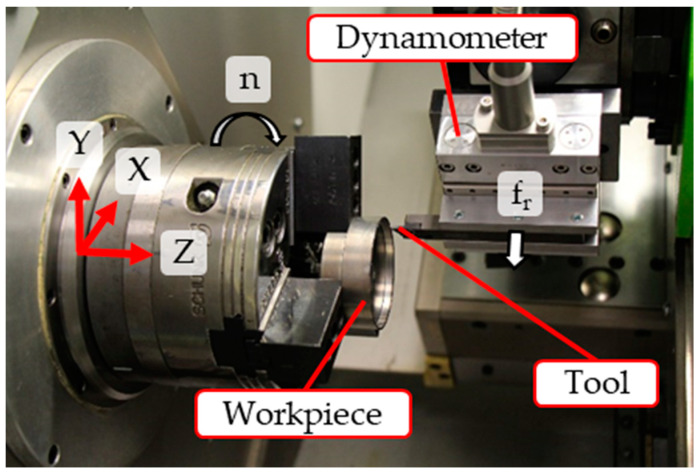
The kinematics of the machining process.

The precision turning experiments were performed on a Go-Future B2 precision lathe (Carl Benzinger GmbH, Pforzheim, Germany), as displayed in Figure 1. The tool holder was mounted on a dynamometer attached to the tool revolver. This multicomponent dynamometer Type 9119AA1 (Kistler Instrumente GmbH, Sindelfingen, Germany) was used to determine the process forces with an additional charge amplifier 5080A and a data acquisition card NI-USB 6361.

**Figure 2:**
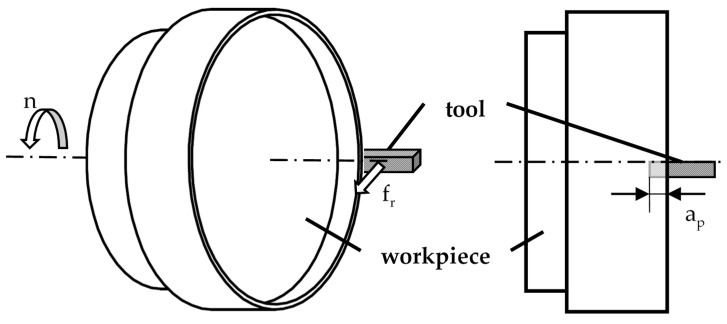
Kinematics of the machining process.

In order to vary material loads for a validation of the corresponding process simulation, the machining parameter a_p_ was varied while the cutting speed was fixed to v_c_ = 100 m/min. In order to ensure a single tool engagement on a certain point of the workpiece material, the feed f_r_ in the radial direction was set to 2.5 mm/rev and therefore exceeds the tool width. One experiment for each parameter combination was performed and, in each experiment, a fresh tool and a new workpiece were used. The machining parameters are summarized in Table 2, while in Figure 2, the kinematics of the machining process is displayed.

### 2.4. Residual Stress Analysis

To determine the material state after the process, X-ray diffraction techniques provide insight into the residual stress state and crystalline structure. The standard sin²Ψ-method [10] was used to analyze the residual stress state in two orthogonal directions on the surface and with respect to depth using material removal by electropolishing on the outer ring. All measurements for the different processes were performed using a common template, which is shown in Table 3.

### 2.5. Modelling and Simulation

A previously validated thermo-visco-plastic material model was used in the process simulation. The following sections describe the methods and models used.

#### 2.5.1. Material Model

The material behavior in the thermo-visco-plastic material model included isotropic hardening according to the Johnson–Cook model (JC). Values were taken from the literature [11,12] for thermo-physical and mechanical properties of all materials and are summarized in Table 4 and Table 5. The resulting yield stress in the JC model is given by

(1) $$\bar{\sigma }=\left[A+B\varepsilon_{p}^{n}\right]\left[1+C\;ln\left(\frac{\dot{\varepsilon }}{{\dot{\varepsilon }}_{0}}\right)\right]\left[1-{\left(\frac{T-T_{0}}{T_{m}-T_{0}}\right)}^{m}\right].$$

The parameters of the material are *A*, *B*, *C*, *m* and *n*, with ${\dot{\varepsilon }}_{0}$ as the reference strain rate, $T_{m}$ as the melting temperature and $T_{0}$ as the room temperature.

#### 2.5.2. Friction Model

Since the pressure at the contact surface between the cutting tool and the workpiece is very high, there is no constant coefficient of friction for the interaction. Instead, the friction properties at the tool-to-chip interface based on the friction model by Zorev [13] were defined as follows:

(2) $$\tau =\min\;(\tau_{Y},\mu \bar{\sigma })$$

(3) $$\tau_{Y}=\bar{\sigma }/\sqrt{3}$$

In addition to this equation, the shear stress τ is limited either by the shear flow stress $\tau_{Y}$ of the material or by Columb friction behavior with a friction coefficient of *μ* = 0.6 [14]. Table 6 lists the friction model parameters.

#### 2.5.3. Boundary Conditions and Mesh

**Figure 3:**
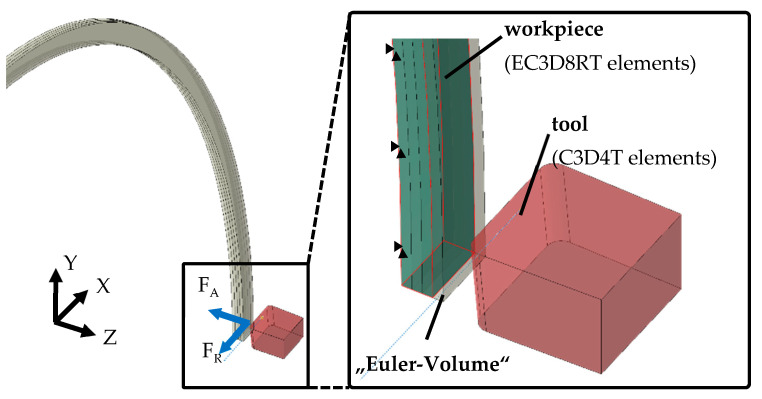
The schematic representation of the cutting tool and workpiece at the start of the engagement in the cutting process.

The cutting tool moves in Euler space during cutting, while the workpiece is fixed in space. A reference point on the axis of rotation is connected to the cutting tool, with all boundary conditions, such as rotation, applied to the reference point. Figure 3 shows the Euler volume and the orientation of the workpiece and tool in space.

In order to model the thermal and mechanical behavior of the workpiece, temperature displacement solid continuum elements were used. The tool was meshed with C3D4T-Lagrange tetrahedral elements, while the Euler-volume meshing was realized with EC3D8RT elements with reduced integration and hourglass control. In [15], Ducobu et al. have studied the influence of the mesh on the results of a CEL orthogonal cutting model. They variated the element size, element orientation and element width. Good results were obtained for 5 µm square elements. Larger elements with a length of 10 µm could be used to obtain faster results with a lower accuracy. Based on the recommendations given in [15] and in our own preliminary investigations, an element size of 7 µm was chosen for the Euler volume in the first 300 µm below the workpiece surface. In order to reduce simulation time, a mesh below the fine mesh with an element size of 50 µm was deployed. The initial temperatures of the workpiece and tool were set to 20 °C (room temperature). Movements of the workpiece were avoided since it was fixed in all directions (displacement along the *X*-axis, *Y*-axis and *Z*-axis and rotation about the *X*-axis, *Y*-axis and *Z*-axis).

In the simulations, a depth of cut a_p1_ = 7 µm and a_p2_ = 150 µm and a cutting velocity of *v_c_* = 100 m/min were chosen.

#### 2.5.4. Mass Scaling of Workpiece for Computational Efficiency

The three-dimensional Eulerian FE analysis is computationally intensive and time consuming. In order to reduce computation time, the mass scaling technique is often applied. In addition to mass scaling, time scaling can be used to further reduce computation time in coupled Eulerian–Lagrangian modeling [16].

The thermal time constant must be maintained in mass as well as in time scaling by adjusting the thermal properties of the material accordingly. The mass is scaled by replacing the density *ρ* with the fictitious density ρ* (*k_m_* > 1).

(4) $$\rho^{*}=k_{m}\rho$$

A replacement of the density *ρ* by the fictitious density ρ* results in a change in the thermal time constant. The fictitious specific heat c_p_^*^ must be used to counter this effect.

(5) $$c_{p}^{*}=c_{p}k_{m}^{-1}$$

A good agreement with experimental values of mechanical forces was achieved with a mass scaling factor of *k_m_* = 25. Furthermore, increasing the mass scaling factor leads to an increase in deviation from experimental results. The computation time at a scaling factor of 25 was approximately 18 h. The factor in this work is similar to the optimal scaling factor for milling and fly-cutting simulations determined in [5,17].

## 3. Results

### 3.1. Measurement Results

#### 3.1.1. Process Forces

**Figure 4:**
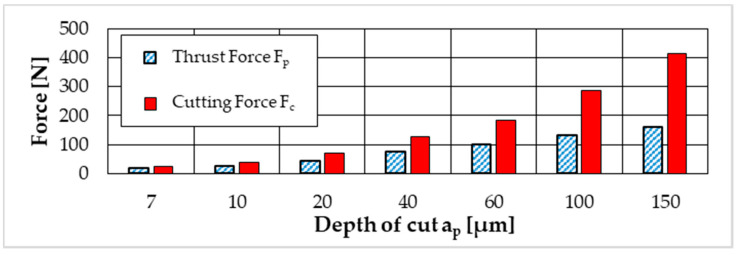
Measured cutting force F_C_ and thrust force F_P_.

The occurring process forces were measured with the dynamometer in the machining experiments. Due to the machining kinematics, the process forces slowly increase with the increase in the width of a cut, resulting in a plateau with the tool fully engaged. Afterwards, the forces slowly decrease as the tool exits the workpiece on the inner side of the ring. The forces used for comparison with the simulation were taken from the plateau. As expected, the cutting force F_c_ as well as the thrust force F_p_ increases with a greater cutting depth a_p_. The sampling rate in the measurement equipment was set to 20 kHz. The results are shown in Figure 4.

#### 3.1.2. Residual Stresses

**Figure 5:**
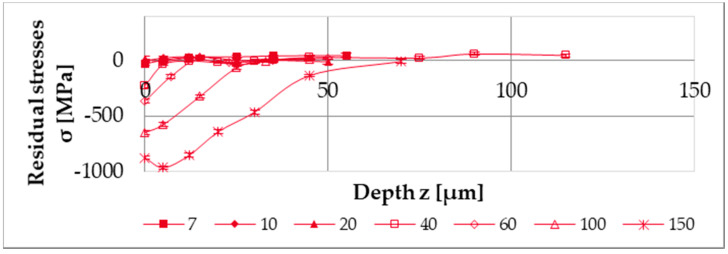
The measured residual stresses parallel to the cutting speed direction.

The resulting material modifications represented by the residual stresses were measured after machining with different cutting depths a_p_. The azimuthal measurement position was located where the 1 mm wide ring was completely machined, i.e., with full engagement of the tool. The residual stresses were determined at different depths, with the results being displayed in Figure 5. A greater depth of cut a_p_ results in higher compressive residual stresses with a higher depth impact.

### 3.2. Simulation Results

#### 3.2.1. Process Forces

In order to validate the process simulation, the measured process forces were compared with the simulation. In order to compare the simulation with the experimental data, the forces operating on the tool were analyzed. As samples for validation, the process was simulated with depth of cuts a_p_ of 7 µm and 150 µm. In conclusion, a maximum difference of 8% between the measured and simulated process forces offers a sufficient parity to further analyze local material loads within the simulation.

#### 3.2.2. Material Loads

**Figure 6:**
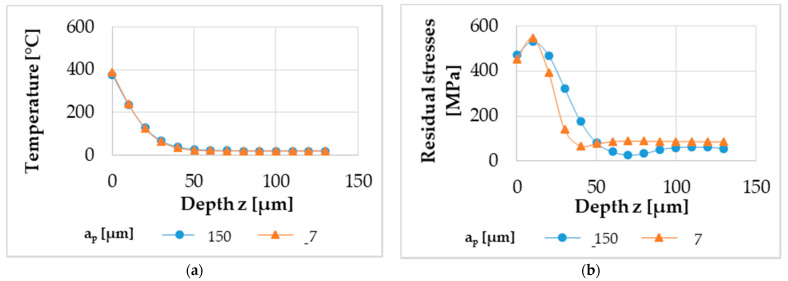
The material loads of local temperature (**a**) and local von-Mises-stresses (**b**) determined in the process simulation.

The material model, which was also utilized and validated in [5], was used in the process simulation. Although no temperatures were measured during the machining experiments described in this article, the material model is proven to accurately determine local material load in the form of temperatures in previous temperature measurements [5]. However, measured forces were used to validate the material model and process simulation regarding the local material load in terms of von-Mises-stresses. The resolution in the simulation is much higher compared to experimental measurements due to sensor sizes or reachability of the measurement area. During the machining with single tool engagement, the maximum temperature and von-Mises-stresses were determined for each volume element. For further processing, a single stack of volume elements in the middle of the 1 mm wide ring was analyzed. These data, who are provided in Supplementary Material S1, result in a graph for temperature and von-Mises-stresses at different depths from the surface z and are shown in Figure 6a,b for cutting depths a_p_ of 7 µm and 150 µm, respectively. Visual representations are given in supplementary data S2 and S3.

## 4. Discussion

**Figure 7:**
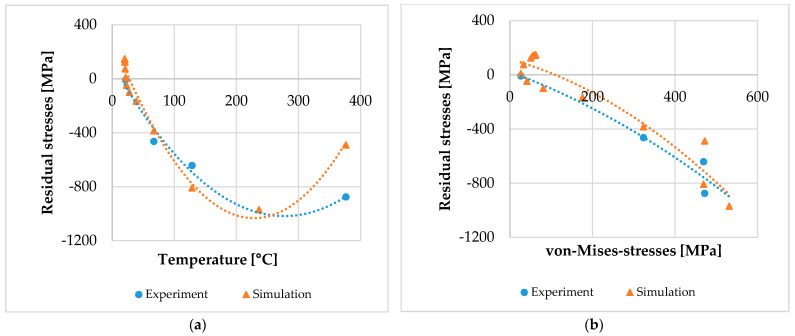
The correlation of experimental data: (**a**) correlation between local temperature and resulting residual stresses; (**b**) correlation between local von-Mises-stresses and resulting residual stresses.

After determining the material loads and material modifications in a much higher resolution compared to the experimental measurements described above, a correlation between material loads and the material modification of the residual stresses was achieved. This correlation considering the local material loads and material modifications is a novel approach investigated in SFB TRR 136 to analyze and understand machining processes as well as the underlying mechanisms. With the data at different depths z of the residual stresses and the material loads, this correlation can be established independently from the depth of the considered volume element. Figure 7a shows the correlation of the residual stresses with the maximum temperature that occurred during machining, while Figure 7b shows the correlation with the maximum von-Mises-stresses that occurred during machining, respectively.

**Figure 8:**
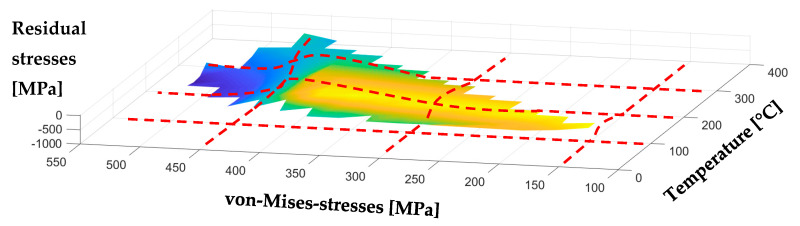
An interpolated three-dimensional representation of local material modification (here: residual stresses) over mechanical and thermal material loads (here: von-Mises-stresses and temperature).

Per definition, these graphs visualize process signature components as they describe the correlation between material loads and material modifications. However, they do not show a pure correlation due to the interdependency of temperature and von-Mises-stresses in the experiments. In other words, high temperatures were only observed with high von-Mises-stresses and vice versa. While these results are a novelty in itself, they do not accommodate the direct correlation that is aimed at. In order to fully understand the influence of the material loads individually, the experimental data and the results achieved numerically must be described with either a constant thermal load or mechanical material load. A process signature, therefore, can only be determined with volume elements exhibiting the same temperature or von-Mises-stresses. Therefore, in an extended approach, the experimental data and the results achieved numerically are interpolated to a three-dimensional graph in supplementary data S4 regarding both material loads. A visual representation is shown in Figure 8.

**Figure 9:**
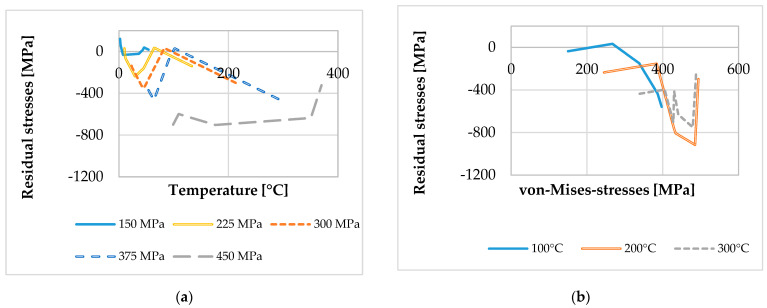
The process signatures: (**a**) correlation between local temperature and resulting residual stresses with constant von-Mises-stresses; (**b**) correlation between local von-Mises-stresses and the resulting residual stresses with constant temperatures.

A mutual representation as shown in Figure 8, which represents the correlation between the maximum temperature and von-Mises-stresses to the residual stresses, was not found in previously published literature. Furthermore, the interpolated data can be cross-sectioned afterwards. A corresponding cross-section of this surface—indicated with red lines in Figure 8—leads to graphs revealing a dependency regarding the material modification of temperature or von-Mises-stresses, while the other material load remains constant. Therefore, process signatures with a correlation with one material load (Figure 9a: temperature; Figure 9b: von-Mises-stresses) are determined with this method.

The newly achieved results indicate that a transformation of material mechanisms occurs and that, with increasing von-Mises-stresses, the overall behavior of the graphs remains the same. The material behavior changes significantly only at the highest mechanical material load with 450 MPa. This change may be addressed by a changing mechanism. Its influence on process signatures will be the subject of further research. Future research will also investigate whether the temperature must be substituted with the temperature gradient as the material load successfully demonstrated in [18]. A possible substitution could be determined by different mechanisms resulting in material modifications.

## 5. Conclusions

In this investigation, machining experiments and a corresponding process simulation were carried out for precision turning of AISI 4140 steel. While the resulting residual stresses were measured by X-ray diffraction, the material loads were determined using a process simulation. The measurement data taken in [5] and machining experiments in this investigation are used to validate the material model and the process simulation. For the material loads, the maximal occurring temperature and the equivalent von-Mises-stresses per volume element during the machining experiments were determined. Furthermore, the experimental data and the results achieved numerically were interpolated and cross sectioned. The resulting correlation between a single material load and the modification of the residual stresses from this investigation based on experiments and simulations is a new contribution to this field. The novel method of a mutual three-dimensional interpolation of two material loads and the corresponding results not only are valuable in the overall scope of the SFB TRR 136 but also enable the prediction of the surface integrity of workpieces. The newly proposed method of representing the material behavior dependent on an individual material load offers potential to identify the different mechanisms in the machining process. The presented process signatures with thermal and mechanical local loads with material modifications offer a great potential to solve the inverse problem of manufacturing.

## Figures and Tables

**Table 1:** The chemical composition of the AISI 4140 steel (in wt. %).

Material	C	Si	Mn	P	S	Cr	Mo
wt. %	0.44	0.26	0.73	0.012	0.002	1.08	0.24

**Table 2:** The machining parameters.

Parameter	Value
Depth of cut a_p_ (µm)	7, 10, 20, 40, 60, 100, 150
Cutting speed v_c_ (m/min)	100
Feed f_r_ (mm/rev)	2.5
Coolant	Dry

**Table 3:** The common template used for X-ray diffraction measurements.

Parameter	Value
Radiation/Filter	Cr-Kα/V
Detector	Line detector
Primary beam	Ø 1 mm
Lattice plane	α{211}
Tube voltage/current	33 kV/40 mA
Ψ angles	11: from 45° to + 45°
Angular step	0.1°
Angular range	147° to 163° in 2θ

**Table 4:** Johnson–Cook constitutive model coefficient for AISI 4140 [11,12].

Coefficient	A (MPa)	B (MPa)	C	m	n	${\dot{\varepsilon }}_{0}\;(s^{-1})$	$T_{m}$ (°C)	$T_{0}$ (°C)
Value	900	650	0.034	0.328	0.45	0.001	1536	20

**Table 5:** The physical and mechanical properties of the workpieces and tools [11,12].

Parameter	Material	Value
E (GPa)	AISI 4140	204
ν	AISI 4140	0.3
ρ (kg/m³)	AISI 4140	7850
	tungsten carbide	15,000
k (W/mK)	AISI 4140	47
	tungsten carbide	46
λ (1/K)	AISI 4140	12.3 × 10^−6^
	tungsten carbide	4.7 × 10^−6^
C_p_ (J/kgK)	AISI 4140	475
	tungsten carbide	203
Inelastic heat fraction		0.9

**Table 6:** The friction model parameters.

Parameter	Value
Columb friction coefficient	0.6
Friction energy converted to heat (%)	100
Fraction of converted heat distributed to tool surface	0.5

## Data Availability

The data presented in this study are partially available in the Supplementary Materials. Additional data presented in this study are available upon request from the corresponding author.

## Supplementary Materials

The following are available online at https://www.mdpi.com/article/10.3390/mi12050526/s1. Measurement Data S1: Measurement data of Figure 5, Figure 6 and Figure 7 as recorded. Figure S2 and S3: Color map of temperature- and von-Mises-stresses distribution taken from simulation. Analysis Data S4: Cross-sectional data for Figure 8.
