# Supplementary figures and images for: An Investigation on Internal Material Loads and Modifications in Precision Turning of Steel 42CrMo4
Source: Micromachines (Basel). 2021 May 6;12(5):526. doi: 10.3390/mi12050526 (PMC8148541; doi:10.3390/mi12050526)

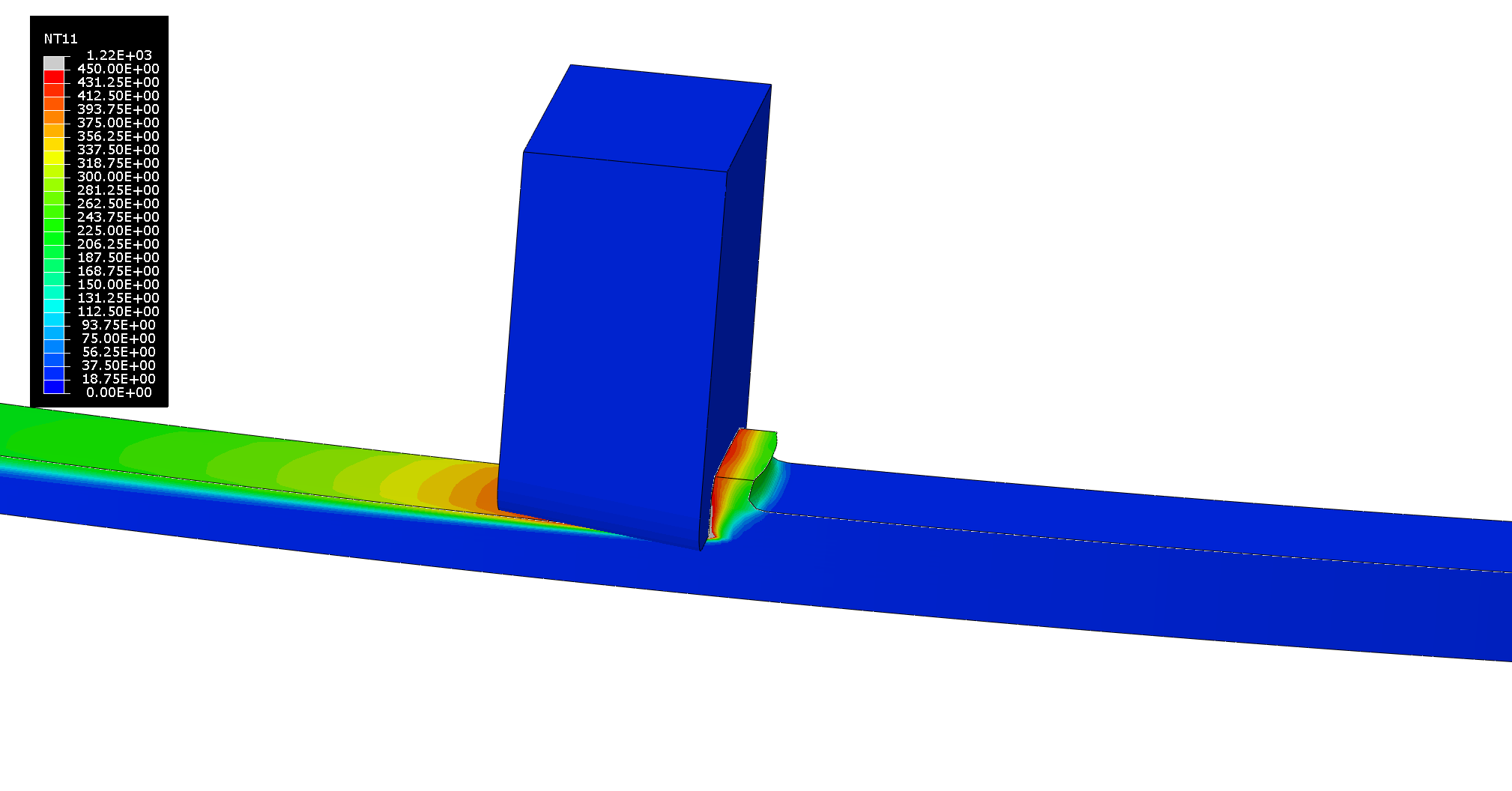

Supplement: Supplementary file 1 [file micromachines-12-00526-s001.zip › 02_Supplementary Data/S2_Color Map_Temperature.png]

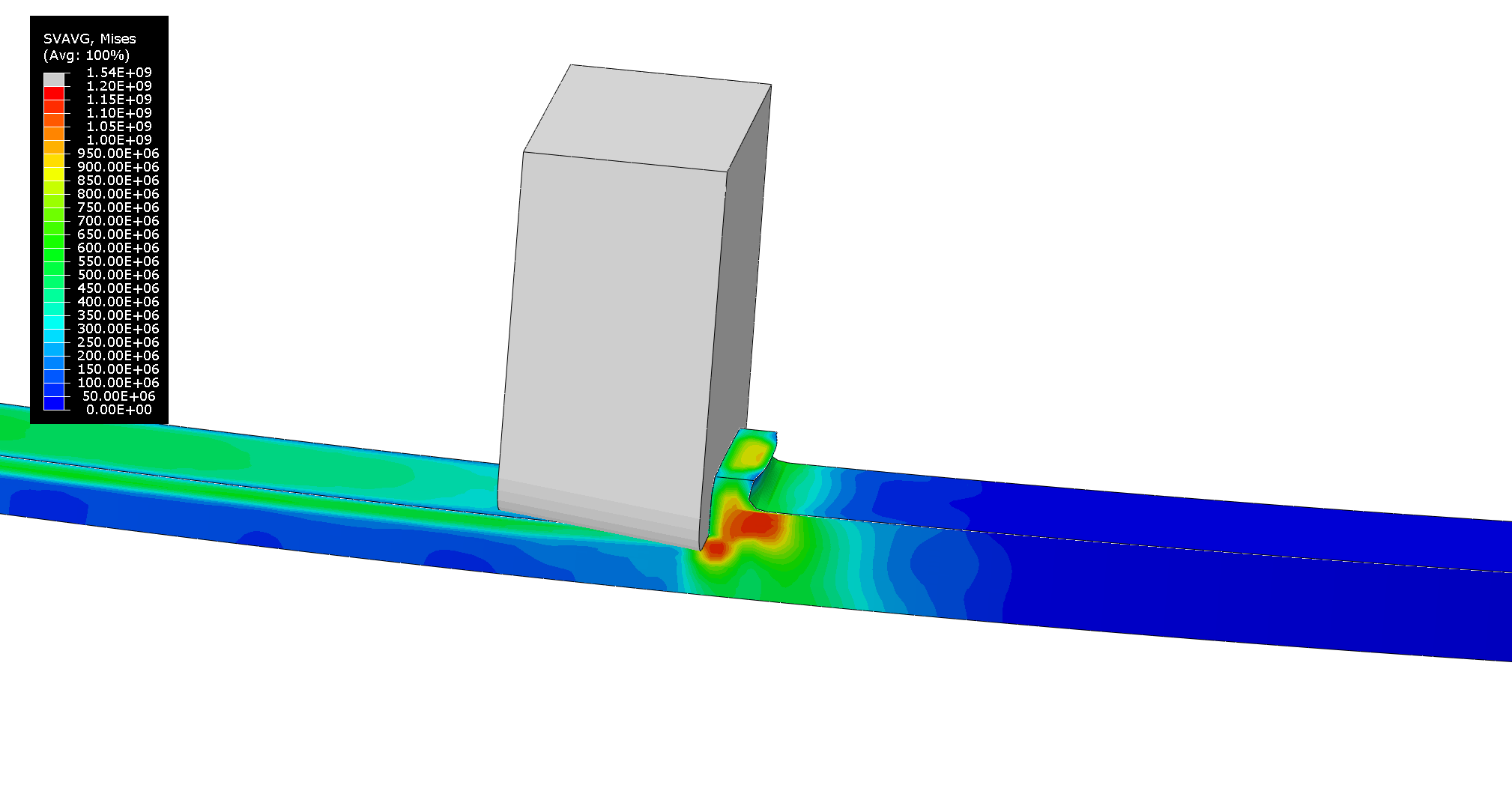

Supplement: Supplementary file 1 [file micromachines-12-00526-s001.zip › 02_Supplementary Data/S3_Color Map_von-Mises-Stresses.png]
